# Tofacitinib and budesonide treatment affect stemness and chemokine release in IBD patient-derived colonoids

**DOI:** 10.1038/s41598-025-86314-2

**Published:** 2025-01-30

**Authors:** Arun Sridhar, Ingunn Bakke, Shreya Gopalakrishnan, Nimo Mukhtar Mohamud Osoble, Emilie Prytz Hammarqvist, Henrik P. Sahlin Pettersen, Arne Kristian Sandvik, Ann Elisabet Østvik, Marianne Doré Hansen, Torunn Bruland

**Affiliations:** 1 Department of Clinical and Molecular Medicine (IKOM), Faculty of Medicine and Health Sciences, NTNU - Norwegian University of Science and Technology, Prinsesse Kristinas gt. 1, Trondheim, 7030 Norway; 2 Department of Gastroenterology and Hepatology, Clinic of Medicine, St. Olav’s University Hospital, Trondheim, Norway; 3 Clinic of Laboratory Medicine, St. Olav’s University Hospital, Trondheim, Norway; 4 Department of Pathology, St. Olav’s University Hospital, Trondheim, Norway

**Keywords:** Intestinal epithelium, Organoids, JAK-inhibitor, Gastrointestinal models, Translational research, Chemokines, Chronic inflammation, Intestinal stem cells, Inflammatory bowel disease

## Abstract

Restoration of the intestinal epithelial barrier is crucial for achieving mucosal healing, the therapeutic goal for inflammatory bowel disease (IBD). During homeostasis, epithelial renewal is maintained by crypt stem cells and progenitors that cease to divide as they differentiate into mature colonocytes. Inflammation is a major effector of mucosal damage in IBD and has been found to affect epithelial stemness, regeneration and cellular functions. However, the impact of immune cell-modulating IBD drugs on epithelial homeostasis and repair is poorly understood. It is likely that these drugs will have distinct mechanisms of action (MOA) in intestinal epithelium relevant for homeostasis that will vary among patients. We investigated cellular effects of pan-Janus Kinase (JAK) inhibitor tofacitinib and the corticosteroid budesonide on uninflamed and TNF + Poly(I:C) stimulated human colon organoids (colonoids) from healthy donors and IBD-patients. Our findings reveal that although both tofacitinib and budesonide exhibit anti-inflammatory effects, tofacitinib increased colonoid size and proliferation during differentiation, and promoted epithelial stemness. In contrast, budesonide decreased colonoid size and showed no consistent effect on proliferation or stemness. Our study demonstrates the value of employing human colonoids to investigate how IBD drugs affect intestinal epithelial cells and inter-individual variations relevant to mucosal healing and personalized IBD treatment.

**Supplementary Information:**

The online version contains supplementary material available at 10.1038/s41598-025-86314-2.

## Introduction

The pathobiology behind chronic relapsing-remitting inflammation in ulcerative colitis (UC) is not fully comprehended. However, it is known that biological mechanisms involve disturbance in the immune system, the intestinal epithelium, and microbiota diversity, particularly in individuals with a genetic predisposition^1^. Intestinal epithelial cells (IECs) separate gut microbiota from immune cells in lamina propria, and disruption of the epithelial barrier is a hallmark of IBD, contributing to inflammation. In addition, IECs secrete chemokines that promote the infiltration of immune cells to lamina propria, thereby driving inflammation further^2^. Mucosal healing, defined as the resolution of inflammation and restoration of the intestinal epithelial barrier, is believed to cause long-term remission in patients treated for IBD. During homeostasis, epithelial renewal is maintained by leucine-rich repeat-containing G-protein coupled receptor 5+ (Lgr5+) intestinal stem cells in the crypts, producing proliferative progenitors that gradually cease to divide as they move towards the surface and differentiate into mature cytokeratin 20 (CK20) expressing colonocytes. Inflammation induces a deviating regenerative program that is incompletely understood but may involve increased capacity of differentiated cells to revert to a more stem-like state with features essential for epithelial repair^3,4^.

The link between IEC growth, chemokine release and cell death are complex and interconnected. Mucosal healing involves epithelial proliferation and differentiation; conversely, disrupting epithelial integrity and behavior can hinder healing. To date, drugs have not been developed to enhance gut barrier function^5^. Conventional IBD treatments include aminosalicylates, immunosuppressants, and corticosteroids, which are first-line therapeutics that reduce inflammation and maintain remission in moderate to severe IBD^6,7^. Budesonide is among the standard steroids with potent anti-inflammatory effects^8^. Recently, therapeutic paradigms have been shifted to biologics and small molecules such as JAK (Janus kinase) inhibitors that target JAK1, JAK2, JAK3, and Tyk2, leading to the blockade of various cytokine receptors and the signal transducer and activator of transcription protein (STAT) pathway^9,10^. Tofacitinib [Xeljanz^®^], the first UC-approved oral pan-JAK inhibitor, modulates the immune response in IBD by inhibiting, e.g., signaling pathways involved in inflammation^11^. Since homeostasis in the intestinal epithelium is critical for achieving and maintaining mucosal healing, we need more knowledge about how these different classes of drugs, primarily developed to modulate immune cell functions in IBD, affect epithelial functions.

Intestinal epithelial organoid models have emerged as invaluable tools for both disease modeling and drug testing in IECs^12^. For example, inhibiting the JAK pathway using tofacitinib or activating the glucocorticoid receptor using steroids have been shown to reduce inflammation-induced barrier dysfunction and cytokine release in IEC lines and human colonic organoids (colonoids)^13–15^. Importantly, intestinal epithelial organoids derived from adult stem cells can recapitulate important features of the intestinal epithelium in a donor-specific manner^16–20^. This study aimed to investigate further the effect of tofacitinib and budesonide directly on IECs, examining growth, stemness, cell death, and chemokine release in both uninflamed and TNF + Poly(I:C) stimulated patient-derived colonoids during differentiation.

## Materials and methods

Materials are listed in Supplementary Table 1.

### Human colonoid culture and experimental design

**Fig. 1:**
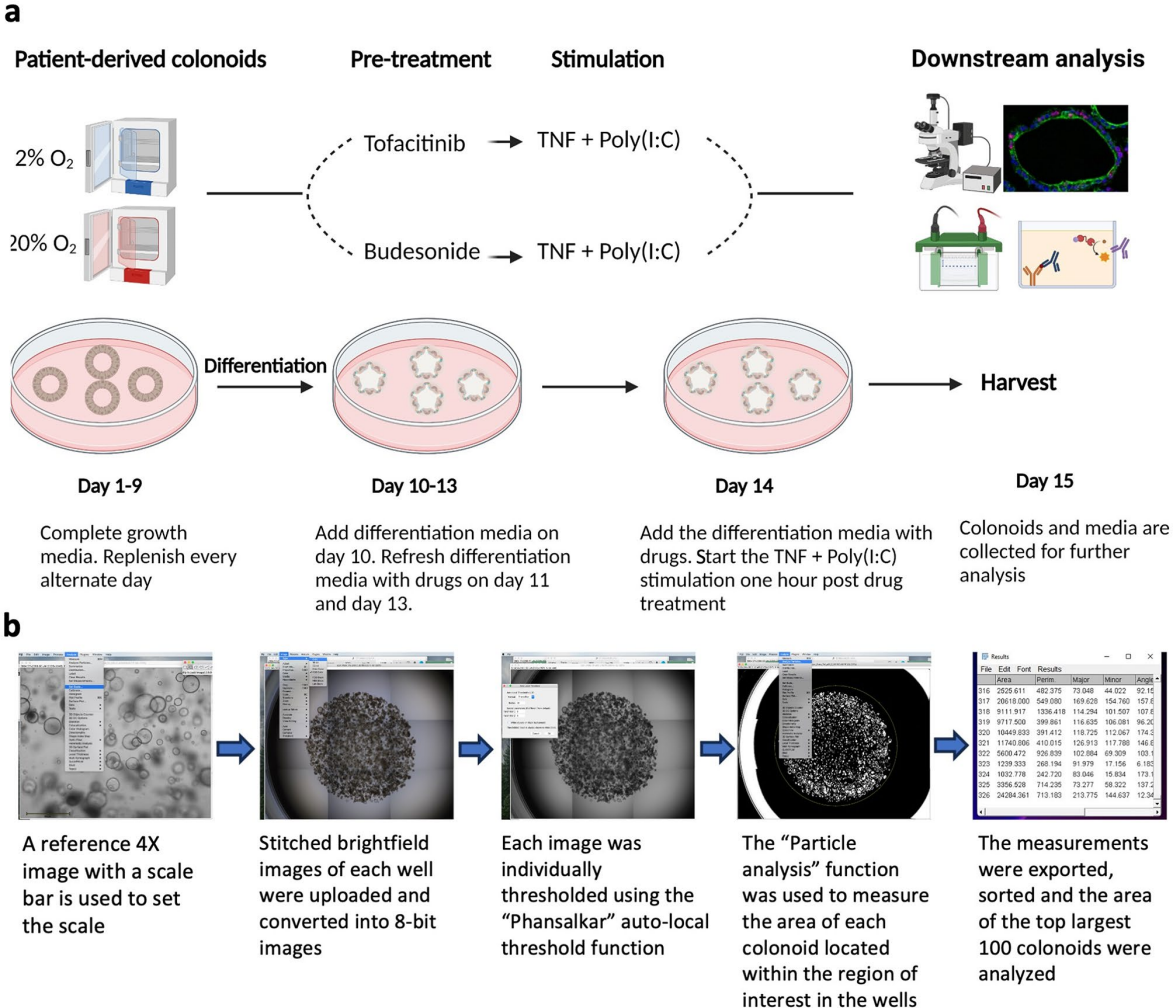
Experimental design and pipeline for colonoid size calculation. (**a**) Graphical overview of experimental design, treatment, and downstream assays. Created with BioRender.com. (**b**) Outline of pipeline used for calculating the area of colonoids using Fiji software^22^.

Intestinal epithelial organoids from human uninflamed colonic biopsies (colonoids) were established, grown, and differentiated as previously described^21^. The patients provided informed written consent under the approval by the Central Norway Regional Committee for Medical and Health Research Ethics (reference numbers 5.2007.910 and 2013/212/REKmidt) and all the experimental methods were performed adhering to the principles of the Declaration of Helsinki. Donor characteristics and a general overview of experimental design in the present work are presented in Table 1; Fig. 1, respectively. In brief, for all experiments the colonoids were dissociated into single cells and resuspended in an ice-cold basement membrane matrix Matrigel GFR (#734–1101, Corning^®^) before 10,000 cells in 50µL Matrigel were plated in each well of prewarmed 24-well plates. For most experiments, colonoids from the same donors were cultured in parallel in two separate incubators at 37^o^C with either 2% or 20% O_2_ with 5% CO_2_, as described^17^. The colonoids were maintained in the complete growth medium for nine days before differentiation was initiated on day ten by lowering the Wnt3A (Wnt3A conditioned medium, #CRL-2647, ATCC) concentration to 5%, withdrawing nicotinamide (#N3376-100G, MerckMillipore) and SB202190 factor (#S7067, Sigma-Aldrich), and adding the pan-Notch inhibitor DAPT (#2634, Bio-Techne) to the medium. For drug treatment, a final concentration of 50µM tofacitinib (Tofacitinib Citrate, 25.2 µg/mL, CP-690550, #S5001, Selleckchem.com), 10µM budesonide (Budesonide, 4.3 µg/mL, # S1286, Selleckchem.com), or 0.033% dimethyl sulfoxide (DMSO, vehicle control) were added in the differentiation media on day 11 and refreshed on day 13 and 1 h before stimulation with a combination of the inflammatory ligands tumor necrosis factor (TNF) (Recombinant human TNF-alpha, 100 ng/mL, #300–01 A, PeproTech) + polyinosinic:polycytidylic acid (Poly(I:C)) (20 µg/mL, #tlrl-pic, InvivoGen) on day 14, as described^19^. On day 15, conditioned media and the colonoids were collected for downstream assays as described below (Fig. 1a).

**Table 1:** Colonoid donor characteristics.

Donor (D)	HC1	HC2	HC3	HC4	HC5	UC1	UC2	UC3
Diagnosis	Non-IBD	Non-IBD	Non-IBD	Non-IBD	Non-IBD	UC	UC	UC
Age	55	28	65	43	43	18	20	29
Sex	Female	Female	Female	Female	Female	Male	Male	Female
Medication								
5ASA	N/A	N/A	N/A	N/A	N/A	1	1	1
Steroid	N/A	N/A	N/A	N/A	N/A	1	0	1
Growth analysis Figs. 2 and 3	x	x	x	x	x	x	x	x
Ki67 and CK20 staining Fig. 4	x	x	x			x	x	x
Western blot Figs. 5 and 7	x	x		x	x		x	x
TUNEL Fig. 6	x	x	x			x	x	x
Multiplex and ELISA Fig. 8	x	x	x			x	x	x

All colonic biopsies used to generate colonoids were taken from uninflamed, right (hepatic) flexure.

IBD-Inflammatory bowel disease, HC-Healthy control, UC-Ulcerative colitis, 5ASA-5-aminosalicylic acid.

### Brightfield image analysis and size quantification of colonoids

Colonoids from 8 donors (Table 1) were cultured at 2% and 20% oxygen concentrations and treated with tofacitinib or budesonide. Two independent experiments were performed for 7 of the donors and 1 experiment for 1 donor. Colonoids were cultured in 3–6 wells for each experimental condition as technical replicates. On day 15, brightfield 4X images (9–12) were taken from each well using an EVOS microscope (Thermo Fischer Scientific) and stitched to an image covering the whole well. All the stitched brightfield images were analyzed in silico through Fiji (RRID: SCR_002285)^22^ by converting into 8-bit images and using the auto local threshold function “Phansalkar” radius to threshold the images. A different Phansalkar radius was used for each independent experiment of donors to capture the inter-individual difference. A region of interest was selected around the Matrigel-covered part of each image, and the “Particle analysis” function was used to measure the size of each colonoid located within the region of interest in the wells. The settings for the particle analysis function included measuring particles from “10µm to infinity”, “excluding particles at the edges,” and “filling in holes.” To standardize the analysis across all conditions and account for the heterogeneity in colonoids development and structure, we systematically calculated average size (area) of the top 100 largest organoids in each well, in total, 600–1200 colonoids per condition for each donor at 2% and 20% oxygen (Fig. 1b and Supplementary File SF1).

### Immunostaining, in situ cell death detection (TUNEL), and imaging of colonoids

On day 15, colonoids from 3 to 6 wells were pooled for each experimental condition and resuspended in 50 µL Richard-Allan Scientific™ HistoGel™ Specimen Processing Gel (#HG-4000-012, Thermo Fisher Scientific), formalin-fixed for 24 h and embedded in paraffin as colonoid cell pellets as previously described^21^. The Dako Autostainer Plus (Dako Agilent) and EnVision FLEX+, Mouse, High pH kit (#K8012, Dako Agilent) were used for immunohistochemical staining of Ki67 and CK20. Formalin-fixed paraffin embedded (FFPE) sections (4 μm) were heated at 60 °C for 1 h and pre-treated in a PT Link Pre-Treatment Module for Tissue Specimens (Dako Agilent) with high pH buffer (pH 9.0) at 97 °C for 20 min and cooled to 65 °C. The sections were transferred to the Dako Autostainer at room temperature and blocked for endogenous peroxidase for 5 min, incubated with the primary antibodies against Ki67 (1:600, #M7240, Dako Agilent) or CK20 (1:1000, #M7019, Dako Agilent) for 2 h, treated with mouse linker for 15 min and 3, 3’-diaminobenzidine (DAB) for 5 min before manual mounting. All sections were scanned with NanoZoomer S360 (Hamamatsu, Japan) with 40X resolution. The whole slide images were quantified using the open-source software QuPath 0.3.2 (RRID: SCR_018257)^23^, as described below.

For double immunofluorescence staining of Ki67 and CK20 and cell death detection, FFPE cell pellet sections (4 μm) were de-paraffinized and rehydrated with Neo-clear, a graded series of ethanol and water. To detect co-localization of Ki67 and CK20, the MaxDouble M488&R650 ImmunoFluorescence Double Staining Kit for human tissue (#DSMR-H3, MaxVision Biosciences Inc.) was used according to the manufacturer’s instructions. Antigen retrieval was performed in boiling Tris-EDTA buffer (pH 9.0), and incubation with the two primary antibodies was done simultaneously for 2 h at room temperature with dilutions 1:50 (#M7240, a-KI67) and 1:150 (M#M7019, a-CK20), and 4’,6-diamidino-2-phenylindole (DAPI) was used as counterstaining. Confocal microscopy (LSM 880 Airyscan, ZEISS, Germany) was used for images captured at 20X magnification with standardized settings normalized to the section with the highest immunofluorescent expression (Supplementary File SF2). For each section, a tile scan with only DAPI was taken of the whole colonoid pellet at 20X magnification, and four random spots of colonoids were chosen within each scan for further quantification as described below. To detect cell death, the terminal deoxynucleotidyl transferase-mediated dUTP nick end labeling (TUNEL) assay (#11684795910, In Situ Cell Death Detection Kit, Fluorescein, Roche) was used according to the manufacturer’s instructions. FFPE sections of the colonoid pellets were placed in a humidified chamber at 37^o^C and pretreated with Proteinase K (#AM2546, Thermo Fisher Scientific, 20 µg/ml diluted in 10mM Tris-HCL) for 30 min, before incubation with TUNEL reaction mixture for 60 min. DAPI was used as counterstaining, and the sections were mounted. Positive fluorescent signal was detected with confocal microscopy (LSM 880 Airyscan, ZEISS) captured at 20X magnification with standardized settings normalized to the section with the highest immunofluorescent expression (Supplementary File SF2). For each section, four to six images covering most of the colonoid pellets were quantified as described below.

### Automated estimation of the CK20 positive area in colonoids

The color deconvolution of the CK20 stain was manually set for a representative image scan. Post-image deconvolution (hematoxylin/DAB), a QuPath pixel classifier was trained to distinguish colonoid structures from the background and create the detection objects. Subsequently, these detections were subjected to shape and intensity measurements, and unwanted objects were further excluded by OD Sum (cut off 0.15), solidity (cut off 0.25), and the presence of residual color (cut off 0). After converting the remaining colonoid detections to annotations, CK20 positive areas within the total colonoid annotations were created using a single measurement thresholder with a setting of DAB mean 0.3 with a sigma of 2.5. The percentage area of CK20 positive cells per total area of cells on each section was quantified for colonoids from 3 HC and 3 UC donors that were grown at both 2% and 20% oxygen condition and treated with DMSO (control), tofacitinib or budesonide. Script and quantifications are provided in Supplementary File SF3.

### Automated estimation of Ki67 positive cell nuclei in colonoids

The color deconvolution of the Ki67 stain was manually set for a representative image scan. A separate QuPath pixel classifier was trained to distinguish colonoid structures from the background for the Ki67 stained images. Unwanted objects were further excluded by measurements similar to the CK20 colonoid detection refinements as described above. All colonoid detections were then converted to annotations. For the Ki67 positive cell detection, we implemented the ‘PositiveCellDetection’ plugin from QuPath, using the hematoxylin deconvolved channel. The settings were adjusted to ensure best possible recognition of Ki67 positive nuclei, with parameters optimally adjusted; “requestedPixelSizeMicrons”: 0.5, “backgroundRadiusMicrons”: 8.0, “medianRadiusMicrons”: 0.0, “sigmaMicrons”: 1.3, “minAreaMicrons”: 15.0, “maxAreaMicrons”: 200.0 and “threshold”: 0.1. Nuclei with hematoxylin intensity less than 0.11 were excluded by a single measurement classifier. Nuclei with Ki67 DAB OD mean greater than 0.15 were classified as positive. The percentage of Ki67 positive cells per total number of cells on each section was quantified for colonoids from 3 HC and 3 UC donors that were grown at both 2% and 20% oxygen condition and treated with DMSO (control), tofacitinib or budesonide. Script and quantifications are provided in Supplementary File SF4.

### Automated quantification of triple positive DAPI, Ki67, and CK20 cells in colonoids

The initial steps were identical to the automated quantification of TUNEL Positive Areas using StarDist as described below, except that cell nucleus expansion (simulated cytoplasm) was included, and cells were excluded if their mean signal in the DAPI channel (nucleus) fell beneath a threshold of 25. The Ki67 (nucleus) and CK20 (cytoplasm) mean intensity was measured for all remaining StarDist detections. A positive cut-off at 15 for the CK20 channel and a positive cut-off at 25 for the Ki67 channel was created as a simple measurements classifier and combined with a compound measurement classifier. Thus, from this, the number of Ki67 positive cells in both CK20 positive cells and CK20 negative cells could be obtained. The percentage of Ki67 positive cells within the total number of CK20 positive cells on each section was quantified for colonoids from 3 HC and 3 UC donors that were grown at both 2% and 20% oxygen condition and treated with DMSO (control), tofacitinib or budesonide. Script and quantifications are provided in Supplementary File SF5.

### Automated quantification of TUNEL positive areas in colonoids

For the quantification of TUNEL-positive regions within the colonoid cell pellets, the StarDist algorithm was integrated via QuPath scripting^24^. Using the StarDist model within QuPath, the specific pre-trained model dsb2018 paper.pb was invoked. Key settings for the StarDist detection included detection probability threshold 0.6, channel selection ‘Ch1-T2’ (DAPI), and requested pixel size 0.225 μm. Post detection, nuclei were excluded if the area was below 12 μm or above 175 μm or if the mean DAPI channel signal was below 60. After that, annotations were generated based on a pixel classifier for areas with a green channel mean signal above 68.2. Detected objects were converted into annotations, and shape simplification was applied (threshold 1.5) using QuPath’s Shape Simplifier, which was then converted back to detections. Then, the number of DAPI-positive StarDist detections and green color channel-positive objects were obtained. The area of TUNEL-positive cells per total number of DAPI cells on each section was quantified for colonoids from 3 HC and 3 UC donors that were grown at both 2% and 20% oxygen condition and treated with DMSO (control), tofacitinib or budesonide. Script and quantifications are provided in Supplementary File SF6.

### Immunoblotting

Colonoids were collected on day 15 for immunoblotting as described previously^21^ and stored at -80 °C until further use. Proteins were isolated from the colonoids by lysing for 2 h on ice using lysis buffer containing 50 mM Tris-HCl pH 7.5, 150 mM NaCl, 5 mM EDTA (#15575-038, Invitrogen), 1% NP-40 (#492018, Sigma-Aldrich), 1 mM dithiothreitol, 1x Complete^®^ EDTA-free protease inhibitor (#11873580001, Sigma-Aldrich), and 1x phosphatase inhibitor cocktail II (#P5726, Sigma-Aldrich) and III (#P0044, Sigma-Aldrich), respectively. Cell lysates were clarified by centrifugation at 13,000 × g for 20 min at 4 °C, and protein concentration was measured using the Pierce BCA protein assay (#23225, Thermo Fisher Scientific). Protein lysates were then denatured in 1 × NuPage lithium dodecyl sulfate sample buffer supplemented with 50 mM dithiothreitol for 10 min at 70 °C before they were separated on 4–12% NuPage Bis-Tris gels (# NP0321BOX, Invitrogen) and electroblotted onto nitrocellulose membranes (0.2 μm, 1704158, Bio-Rad) using the Trans-Blot Turbo Transfer System (Bio-Rad). Membranes were blocked using Rockland Blocking Buffer for Fluorescent Western Blotting (#MB-070, Rockland) for 1 h at room temperature before incubation with the indicated antibodies overnight at 4 °C. The blots were incubated with Dylight secondary antibodies (Invitrogen) for visualization (Supplementary Table 1). Images were obtained with LI-COR Odyssey Imager and analyzed using Image Studio Software (LI‐COR Biosciences, NE, USA). Total protein levels were normalized to glyceraldehyde-3-phosphate dehydrogenase (GAPDH) expression and given as fold change relative to untreated DMSO control (Supplementary File SF7). The following antibodies were used: LGR5 Mouse mAb (1:1000, #MA5-25644, Invitrogen), EPH Receptor B2 (EphB2) Goat mAb (1:1000 dilution, #AF467, R&D Systems), Phospho-MLKL (Ser358) (E7G7P) Rabbit mAb (1:1000 dilution, #18640, Cell Signaling Technology), MLKL (E7V4W) Mouse mAb (1:1000 dilution, #26539, Cell Signaling Technology), Caspase-3/cleaved caspase-3 (D3R6Y) Rabbit mAb (1:1000 dilution, #14220, Cell Signaling Technology), and GAPDH (D16H11) XP rabbit monoclonal antibody (1:5000 dilution, #5174, Cell Signaling Technology).

### Multiplex chemokine profiling and ELISA

The Bio-Plex Pro Human Chemokine Panel, 40-plex (#171AK99MR2, Bio-Rad) was used to analyze undiluted conditioned medium samples from the colonoids experiments according to the manufacturer’s instructions using the Bio-Plex 200 Systems. As per the manufacturer’s protocol, sandwich ELISA kits were used to measure the CXCL2, CXCL5, and CXCL11 chemokine concentrations in the conditioned media. The 96-well plate was coated with respective capture antibodies diluted in phosphate-buffered saline (PBS) and incubated overnight at room temperature. The plates were washed thrice with PBS + 0.05% Tween 20, blocked with Reagent diluent (1% bovine serum albumin in PBS) for one hour before standard, and samples were added to the plates and incubated for two hours at room temperature. All the samples were analyzed in duplicates with a working dilution of 1:50 or 1:100. Then, Streptavidin-HRP (1:40) was added to the plates and the substrate solution for 20 min. The plates were kept in the dark after the streptavidin step. Finally, stop solution (2 N H_2_So_4_) was added, and the plates were immediately analyzed with a microplate reader (iMark, Bio-Rad).

### Statistical analysis

Data from Multiplex analysis was statistically analyzed using R (RRID: SCR_001905) and all the other data was analyzed in GraphPad Prism 9.0 (RRID: SCR_002798). Normality was assumed, and parametric analyses were used. Paired t-tests were done between two groups as indicated. Donor-dependent effects on group level of tofacitinib and budesonide alone or pre-TNF + Poly(I:C) stimulation were analyzed with repeated measure (RM) one-way ANOVA followed by Šídák’s multiple comparisons tests. For multiplex, only chemokines with concentration between 10 pg/ml to upper detection limit were analyzed. A custom function was implemented to preprocess the data, which involved removing asterisks from all values and converting them to numeric format. Out-of-range (OOR) values were handled specifically: ‘OOR <‘ values were replaced with 50% of the minimum detected value. To evaluate the effect of drugs statistically, a linear mixed model (LMM) was employed using the formula: log(Value) ~ Condition + Oxygen + Condition: Oxygen + (1|Donor), where ‘Value’ represents the chemokine concentration, ‘Condition’ and ‘Oxygen’ are fixed effects, ‘Condition: Oxygen’ represents their interaction, and ‘(1|Donor)’ accounts for random effects due to individual donors. The anlysis was performed in R using the Lmer4^25^ and LmerTest^26^ packages. The model’s residuals were checked to ensure they followed a normal distribution. P-value was adjusted using Benjamin-Hochberg method for multiple comparisons testing. The statistics for ELISA results were done on log2 transformed data (Raw data in Supplementary File SF8). Donor-dependent effects on group level of tofacitinib and budesonide alone or pre-TNF + Poly(I:C) stimulation were analyzed with RM one-way ANOVA followed by Šídák’s multiple comparisons tests. Statistical significances are shown with asterisks *P < 0.05, *** *P* < 0.001, **** *P* < 0.0001. Due to the observed large inter-donor differences, all P-values < 0.2 are reported.

### Conference presentation

AS-UEG-2024-01576 titled “THE IMPACT OF TOFACITINIB AND BUDESONIDE ON STEMNESS, CELL DEATH, AND CHEMOKINE RELEASE IN IBD PATIENT-DERIVED COLONOIDS” has been presented as an E-POSTER (PP0367) on UEG Week 2024.

## Results

### Tofacitinib and budesonide treatment during differentiation affect colonoid size

The ability to promote epithelial homeostasis and healing is important for maintaining mucosal integrity in IBD. To investigate if tofacitinib and budesonide could affect the growth of IECs during the differentiation process, we cultured colonoids derived from 8 donors and treated them with tofacitinib or budesonide from day 11 to 15 (Fig. 1a). The colonic epithelium is adapted to thrive in a low oxygen environment and during homeostasis, oxygen levels naturally range from 3% in crypts to < 1% at the surface^27,28^. Previous work from our group has supported the importance of performing colonoid studies in physiological hypoxia (physioxia, 2% oxygen) to better resemble in vivo conditions^16,17^. Nevertheless, most laboratories still culture colonoids at 18–21% oxygen without accounting for the colonic physiological hypoxia. To facilitate comparisons, we also include results from some parallel experiments at 20% oxygen.

**Fig. 2:**
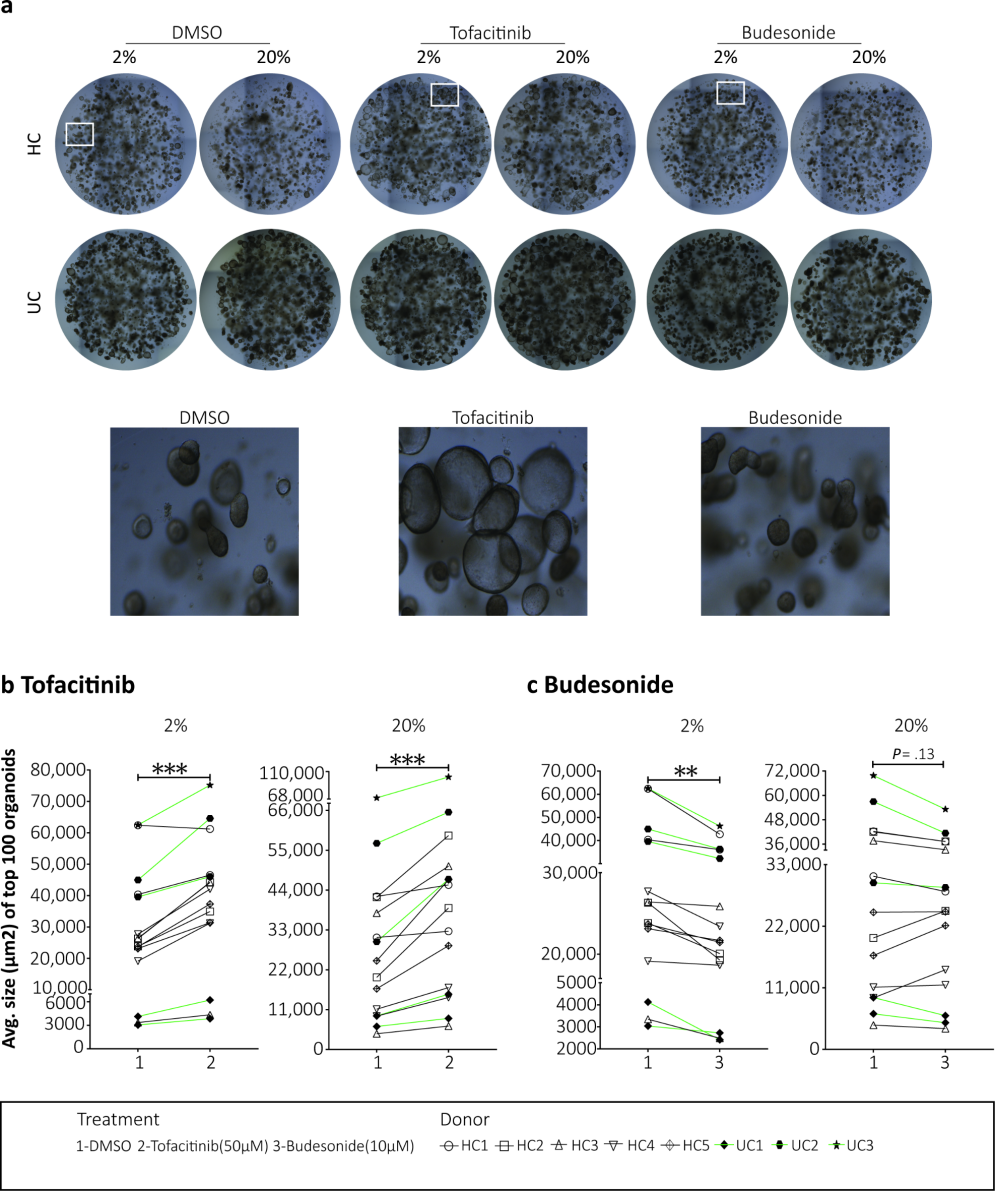
Average colonoid size after tofacitinib and budesonide treatment during differentiation. Brightfield images were captured on day 15 and analyzed using Fiji software^22^ as described in Materials and Methods. (**a**) Representative images of vehicle control (DMSO 0.033%), tofacitinib, and budesonide treated colonoids cultured at 2% or 20% oxygen. The graphs below show the average size of the top 100 colonoids per well (*N* = 3–6) in response to (**b**) tofacitinib and (**c**) budesonide. Independent experiments with colonoids from *N* = 8 donors (5 HC (open shape with black line) and 3 UC (dark shape with green line)) were performed in both 2% and 20% oxygen. Different shape types represent different donors, each with two independent experiments. Differences were evaluated using paired t-test. *P < 0.05, *** *P* < 0.001, **** *P* < 0.0001.

**Fig. 3:**
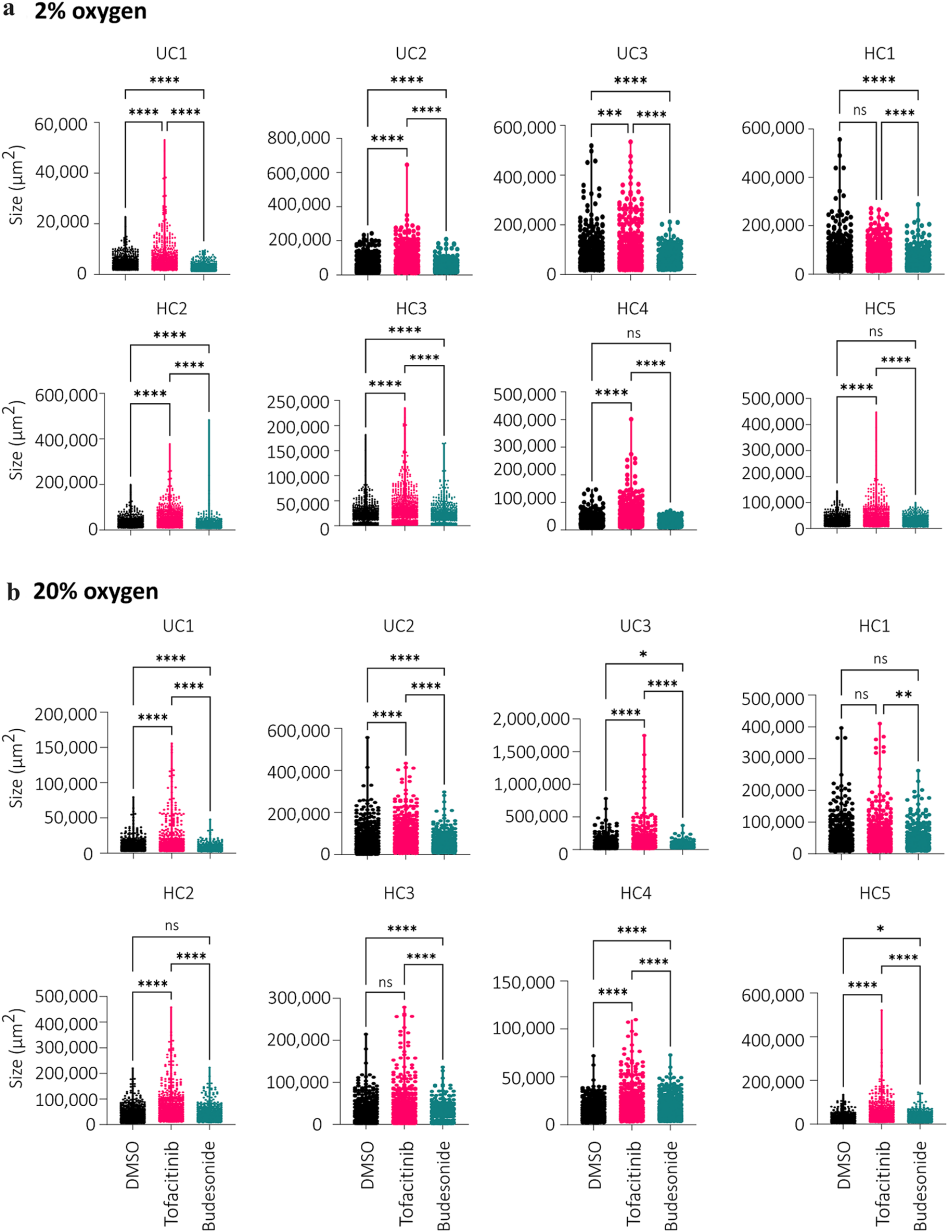
Donor-dependent effects of tofacitinib and budesonide treatment on colonoid size. The violin plots show individual sizes of 600–1200 colonoids in response to vehicle control (DMSO 0.033%), tofacitinib and budesonide treatment for *N* = 8 donors (5HC and 3UC) at (**a**) 2% and (**b**) 20% oxygen. Each dot represents one colonoid. Differences were evaluated using RM one-way ANOVA. *P < 0.05, *** *P* < 0.001, **** *P* < 0.0001.

Brightfield images captured on day 15 were used to analyze size of the largest colonoids in response to the two drugs compared to the DMSO control. Representative images are shown in Fig. 2a. At group level, colonoids treated with tofacitinib had significantly increased average size compared to untreated DMSO controls in both 2% and 20% oxygen (*P* = 0.0002) (Fig. 2b). On the other hand, treatment with budesonide resulted in a significantly reduced average size compared to DMSO controls in 2% oxygen (*P* = 0.003), with a similar trend observed at 20% (*P* = 0.13) (Fig. 2c). We noticed relatively large inter-individual size differences in the colonoids derived from the 8 donors and their response to the two drugs (Fig. 2b-c). To examine this in more detail, we analyzed 600–1200 individual colonoids per treatment for each donor and found that both tofacitinib and budesonide significantly affected the colonoid size in most donors (Supplementary File SF1) grown at both 2% (Fig. 3a) and 20% oxygen (Fig. 3b). The size differences between the colonoids treated with tofacitinib and budesonide were highly significant (*P* = 0.0001) for all donors at both 2 and 20% oxygen (Fig. 3a-b). Overall, when added during the differentiation process, tofacitinib and budesonide had opposing effects on the growth of the colonoids, where tofacitinib led to an increase and budesonide resulted in a decrease in the colonoid size.

### Tofacitinib significantly increases the percentage of terminally differentiated cells with the ability to proliferate

**Fig. 4:**
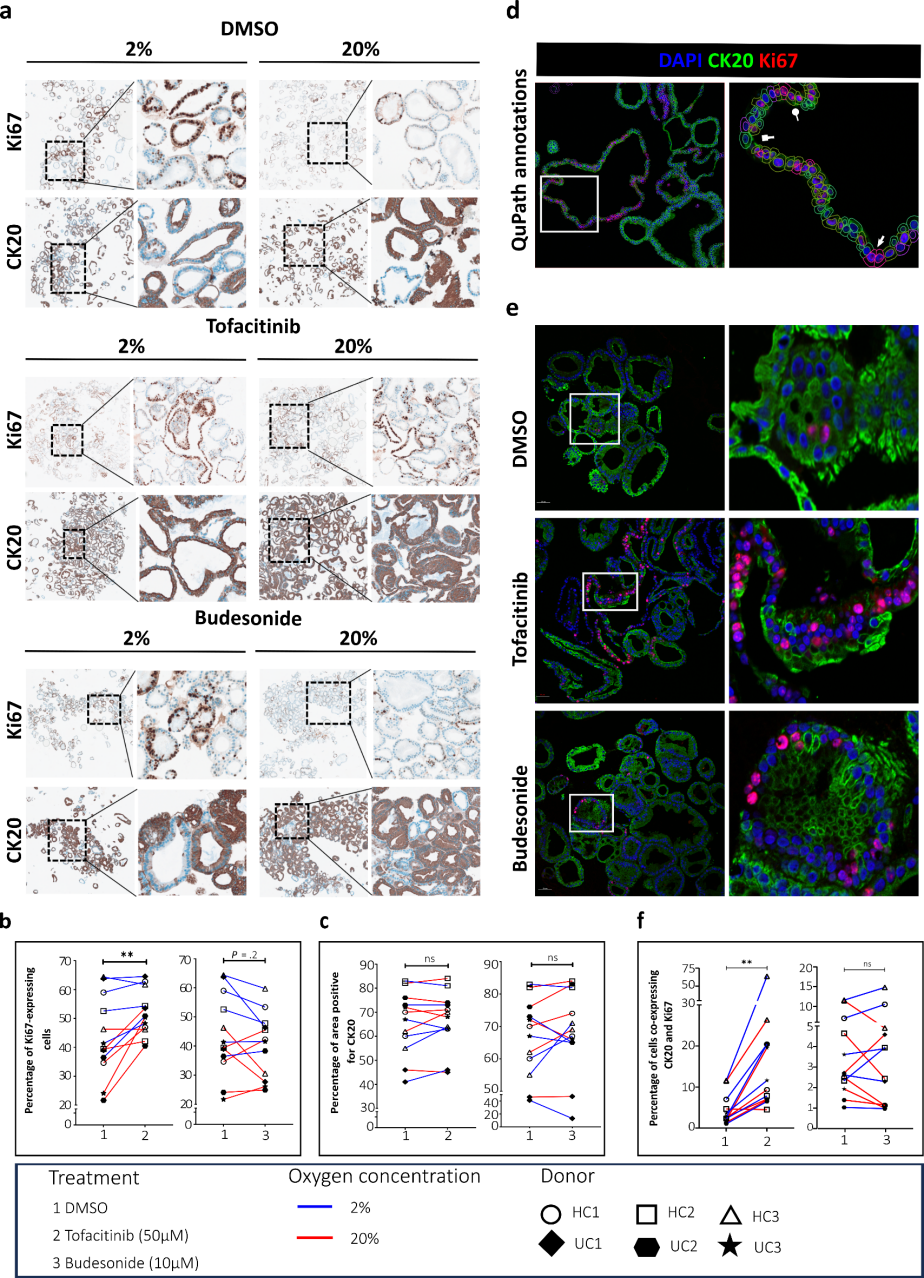
Analysis of CK20 and Ki67 expressions in colonoids in response to tofacitinib or budesonide. (**a**) Representative images of immunohistochemical staining of Ki67 and CK20 protein expression in colonoids from *N* = 6 donors (3HC and 3 UC) in response to vehicle control (DMSO 0.033%), tofacitinib and budesonide cultured at either 2% or 20% oxygen. Graphs showing quantification of the percentage of (**b**) Ki67 expressing cells and (**c**) CK20 positive area in colonoids after treatment with tofacitinib and budesonide compared to vehicle control (DMSO 0.033%) cultured at 2% and 20% oxygen. (**d**) Representative image from the QuPath analysis of CK20 and Ki67 double fluorescence staining. We programmed QuPath to identify the number of single positive Ki67 cells annotated with pink lines (white arrow), single positive CK20 cells annotated with green lines (white squarehead) and Ki67 and CK20 double positive cells annotated with yellow lines (white circlehead). (**e**) Representative images of double fluorescence staining showing co-expression of CK20 and Ki67 in colonoids from *N* = 6 donors (3HC and 3UC) in response to vehicle control (DMSO 0.033%), tofacitinib and budesonide cultured at 2% oxygen. (**f**) Graphs showing quantification of the percentage of cells co-expressing Ki67 and CK20 in colonoids after treatment with tofacitinib and budesonide compared to vehicle control (DMSO 0.033%) cultured at 2% and 20% oxygen. QuPath^23^ (version 0.3.2) software was used for the quantification. Differences evaluated using paired t-test. **P < 0.01.

Since tofacitinib and budesonide impacted colonoid size during differentiation, we further investigated the effect of the drugs on proliferation and differentiation status. Colonoids from 6 donors were stained for the proliferation marker Ki67 and the marker of terminal epithelial maturation in the colon, CK20. Responses to each treatment were computationally analyzed using QuPath^23^. Representative images are shown in Fig. 4a.

Like colonoid size, we observed inter-individual differences in the Ki67- and CK20-expressing cell percentages. In agreement with previously published results^17^, there appeared to be more Ki67-expressing cells in colonoids cultured at 2% compared to 20% oxygen. However, paired analysis showed that tofacitinib increased the percentage of Ki67-expressing cells compared to DMSO control (*P* = 0.008), with similar tendencies for colonoids cultured at 2% (*P* = 0.14) and 20% (*P* = 0.02) oxygen. The Ki67 expression in colonoids was not consistently affected by budesonide treatment (*P* = 0.20) (Fig. 4b) but showed a decrease or no effect in most experiments, especially when cultured at 2% oxygen (*P* = 0.12). The percentage of cells expressing the differentiation marker CK20 was found to be unchanged in colonoids treated with either tofacitinib or budesonide compared to DMSO control when grouping culturing at 2% and 20% oxygen together (Fig. 4c) or looking at either of the oxygen conditions separately.

Next, since tofacitinib increased the proliferation rate without affecting the differentiation of the colonoids, we wondered if the same cells could express both markers. Colonoids were double stained for CK20 and Ki67, and an automated quantification method using QuPath was developed (Fig. 4d). Representative images are shown in Fig. 4e. The results showed that tofacitinib treatment significantly increased the percentage of Ki67-CK20 co-expressing cells in the colonoids cultured at both 2% (*P* = 0.06) and 20% (*P* = 0.03) oxygen and when grouped together (*P* = 0.008) (Fig. 4f). Budesonide treatment had no consistent effect on the percentage of co-expressing cells (Fig. 4f). In summary, tofacitinib treatment during colonoid differentiation was associated with increased Ki67 expression in general and specifically in CK20-expressing cells, indicating that tofacitinib could retain proliferation capacity in fully differentiated cells. Although budesonide treatment reduced colonoid size, the drug did not affect proliferation consistently.

### Stem cell markers are more expressed in tofacitinib-treated colonoids than in budesonide- or untreated colonoids

**Fig. 5:**
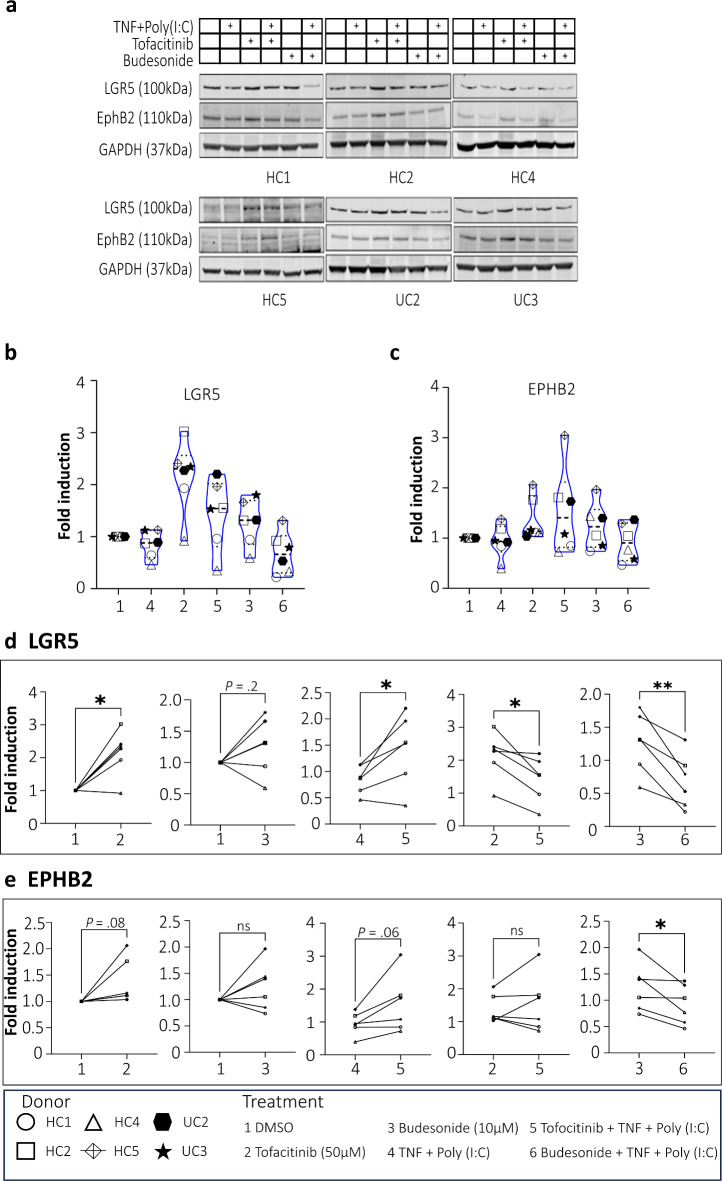
LGR5 and EphB2 protein expression in differentiated colonoids in response to tofacitinib or budesonide treatment. (**a**) Immunoblots showing LGR5 and EphB2 protein expression in colonoids from *N* = 6 donors (4HC and 2UC) cultured at 2% oxygen. Original full blot images are presented in the Supplementary Figure S1. (**b**,**c**) Quantification of LGR5 and EphB2 at group level in response to different treatments (1–6) (**d**,**e**) Paired quantification of LGR5 and EphB2 protein expression between selected treatments. The colonoids were pre-treated with tofacitinib or budesonide for 3 days alone or before TNF + Poly(I:C) (see Fig. 1). The x-axis shows the treatment, and the y-axis shows fold induction. Fold expression is generated by normalizing to vehicle control (DMSO 0.033%) for each donor and further normalized to GAPDH expression. Differences were evaluated using paired t-test. *P < 0.05.

Based on our histomorphological analysis of colonoids revealing increased growth and proliferation after treatment with tofacitinib, we wanted to examine the effect on colonid stemness. Thus, next, we assessed whether tofacitinib and budesonide treatment during differentiation affected protein expression of the intestinal stem cell markers LGR5 and EPHB2^29^. Tofacitinib and budesonide-treated colonoids from 6 donors cultured at 2% oxygen were collected at day 15, and protein extracts were analyzed by immunoblotting (Fig. 5a). After tofacitinib treatment, there was higher levels of LGR5 protein compared to DMSO control in five of the six donors (*P* = 0.01) (Fig. 5b, d). A weaker but similar trend (*P* = 0.08) was seen for EPHB2 (Fig. 5c, e). In budesonide-treated colonoids, we observed, as before, an extensive donor difference and a combination of higher and lower levels of both LGR5 and EPHB2 compared to DMSO control (Fig. 5b-e).

Inflammation is a major effector of intestinal mucosal damage in IBD and has been found to affect epithelial stemness, regeneration, and cellular functions^3,30^. TNF is particularly relevant since TNF-blocking drugs are common in IBD treatment. Toll-like receptor 3 (TLR3) detects double-stranded RNA from damaged tissues and viruses, triggering the release of inflammatory cytokines and potentially promoting cell death^31^. In colonoids, we have shown that the TLR3 ligand Poly(I:C) specifically triggers a type I interferon response^32^ and that tofacitinib downregulates TNF and Poly(I:C)-dependent MHC-II expression^19^. Thus, to examine if the effects of treatment with tofacitinib and budesonide could be altered by inflammatory stimuli, TNF and Poly(I:C) was used to mimic a central IBD cytokine and PPR ligand. We observed that pre-treatment with tofacitinib seemed to retain a higher LGR5 protein expression also when adding TNF + Poly(I:C), compared to TNF + Poly(I:C) stimulation alone (*P* = 0.03) (Fig. 5b, d). This was seen even if adding TNF + Poly(I:C) did reduce the LGR5 levels compared to treatment with tofacitinib alone (*P* = 0.01) (Fig. 5b, d). Also, the EPHB2 protein expression was higher after tofacitinib pre-treatment with TNF + Poly(I:C) stimulation compared to stimulation alone (*P* = 0.06) (Fig. 5c, e). For budesonide pre-treated colonoids, there was again more variation, although both LGR5 and EPHB2 protein levels were lower when adding TNF + Poly(I:C) stimulation to the budesonide treatment compared to budesonide alone (*P* = 0.004 and *P* = 0.04, respectively) (Fig. 5d, e).

Collectively, the results suggest that tofacitinib added during the differentiation process changes the behavior of the IECs by promoting growth, proliferation, and stemness, even under inflammatory conditions. In contrast, despite a general repression, budesonide exhibits an inhibitory effect on colonoid growth with variable and inconsistent effects on the other parameters.

### Tofacitinib and budesonide pre-treatment do not protect against cell death in colonoids

**Fig. 6:**
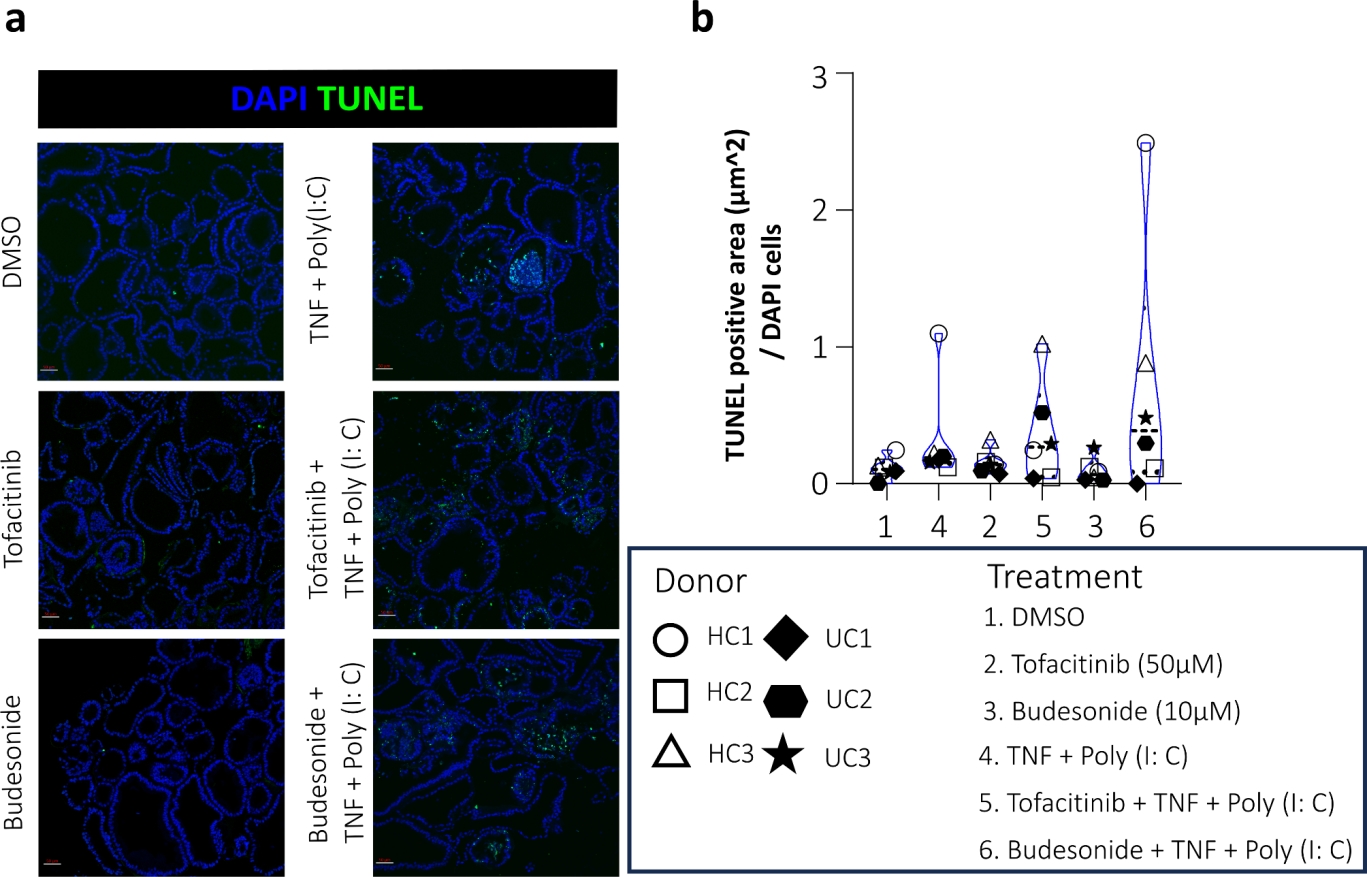
Immunofluorescence staining of differentiated colonoids for cell death (TUNEL assay). (**a**) Representative images of TUNEL labelling illustrate differences in cell death intensity (TUNEL: green fluorescence, DAPI: blue fluorescence) in colonoids from *N* = 6 donors (3HC and 3UC) cultured at 2% oxygen. (**b**) Quantification of TUNEL at group level in response to different treatments (1–6). The colonoids were pre-treated with tofacitinib or budesonide for 3 days alone or before TNF + Poly(I:C) (see Fig. 1). Quantification is shown as TUNEL positive area / number of DAPI-positive cells (µm^2^/cells).

Cell death, such as apoptosis and necroptosis in IECs, is highly regulated, and any irregularities might damage gut homeostasis and mucosal barrier. These cell deaths have been linked to chronic inflammation, which is seen in patients with IBD^33^. Therefore, it was interesting to investigate if tofacitinib and budesonide could alter cell death in the colonoids. First, we looked at apoptotic DNA fragmentation by TUNEL staining of colonoid sections from 6 donors. Representative images are shown in Fig. 6a. The computational immunofluorescence analysis showed that inflammatory stimulation with TNF + Poly(I:C) seemed to be the main factor increasing the number of TUNEL-positive cells. Neither tofacitinib nor budesonide did affect the cell death number alone or reduce TNF + Poly(I:C) induced cell death. (Fig. 6b).

**Fig. 7:**
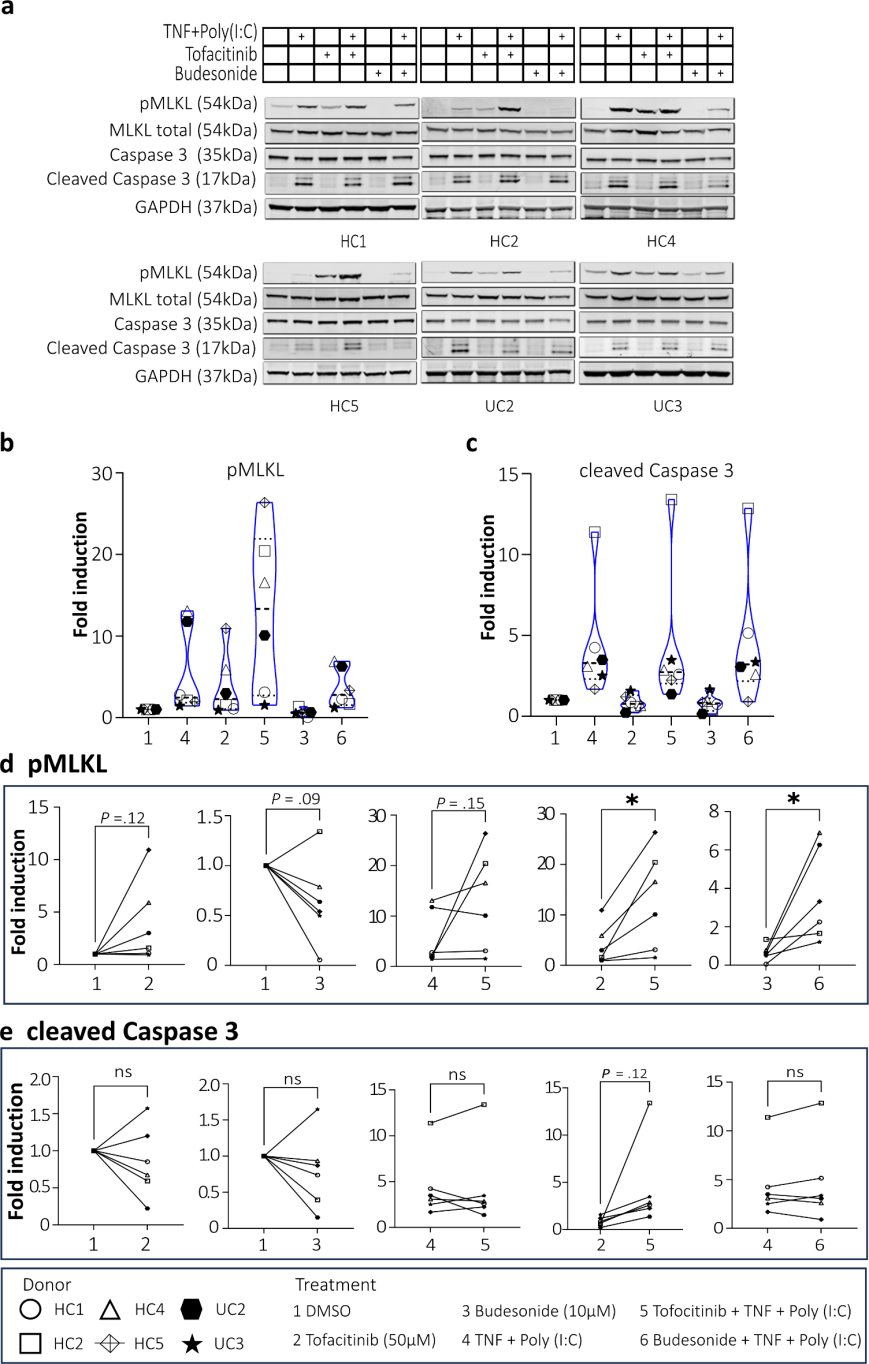
Cell death protein expression in differentiated colonoids in response to tofacitinib or budesonide pre-treatment with or without TNF + Poly(I:C). (**a**) Immunoblots showing pMLKL, MLKL total, caspase 3 and cleaved caspase 3 protein expression in colonoids from *N* = 6 donors (4HC and 2UC) cultured at 2% oxygen. Original full blot images are presented in the Supplementary Figure S2. (**b**, **c**) Quantification of pMLKL/MLKL or cleaved caspase 3/caspase 3 at group level in response to different treatments (1–6) (**d**, **e**) Paired quantification of pMLKL/MLKL or cleaved caspase 3/caspase 3 protein expression between selected treatments. The colonoids were pre-treated with tofacitinib or budesonide for 3 days alone or before TNF + Poly(I :C) (see Fig. 1). The x-axis shows the treatment, and the y-axis shows fold induction. Fold expression is generated by normalizing to vehicle control (0.033% DMSO) for each donor and further normalized to GAPDH expression. Differences were evaluated using paired t-test. *P < 0.05.

We then explored further if we could see an effect of tofacitinib or budesonide on the cell death types MLKL-dependent programmed necrosis (necroptosis) and/or caspase 3-mediated apoptosis by analyzing pMLKL and cleaved caspase 3 by immunoblotting (Fig. 7a). When comparing the different conditions on a group level, there were donor variations and no statistically significant differences (Fig. 7b, c). Treatment with tofacitinib alone during colonoid differentiation tended to induce pMLKL protein expression compared to DMSO control, which in paired analysis was seen in 4 of the 6 donors (*P* = 0.12) (Fig. 7d). Tofacitinib did not show the same trend for cleaved caspase 3 (Fig. 7e). Conversely, budesonide treatment alone slightly decreased protein expression of mainly pMLKL (*P* = 0.09) (Fig. 7d). TNF + Poly(I:C) stimulation alone increased protein expression of both pMLKL (*P* = 0.09) and cleaved caspase 3 (*P* = 0.06) compared to DMSO control. Pre-treating the colonoids with tofacitinib before TNF + Poly(I:C) stimulation further enhanced in the pMLKL expression in some donors compared to tofacitinib alone (*P* = 0.02) and to stimulation alone (*P* = 0.15). Similarly, combining budesonide pre-treatment with TNF + Poly(I:C) stimulation increased the pMLKL protein level compared to budesonide alone (*P* = 0.03) (Fig. 7d). The same trend was not seen for the expression of cleaved caspase 3, where TNF + Poly(I:C) stimulation seemed to be the main driver also in combination with pre-treatment with both drugs. Overall, our analysis indicates that neither tofacitinib nor budesonide pre-treatment during differentiation has definite harmful or protective effects on TNF + Poly(I:C) stimulated cell death in colonoids. Still, we observed large inter-individual variability, especially with tofacitinib, which seems to enhance the expression of the pMLKL necroptosis marker in some donors.

### Tofacitinib and budesonide pre-treatment affects chemokine release from colonoids

**Fig. 8:**
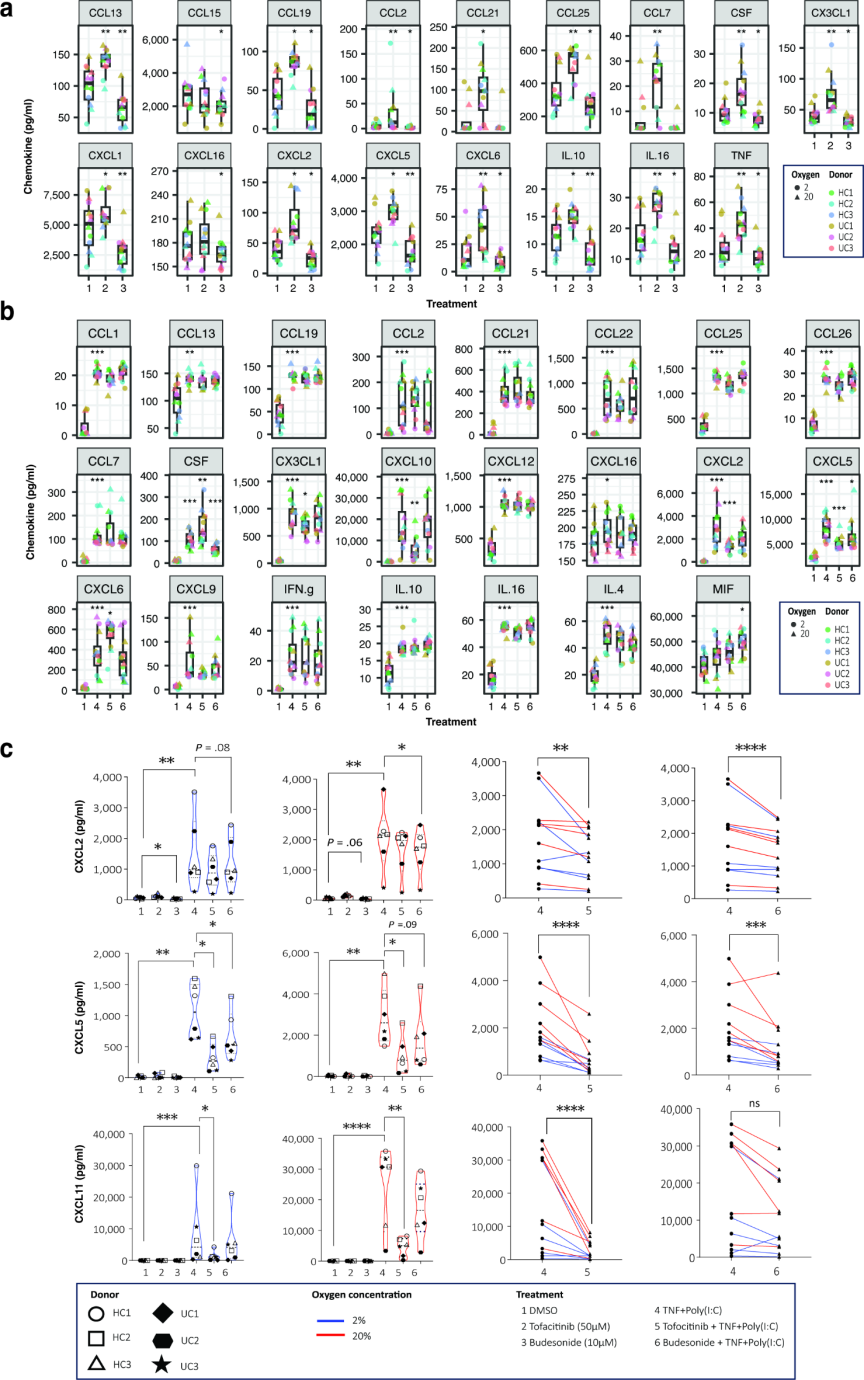
Secretion of chemokines from differentiated colonoids. (**a**,**b**) Detection of chemokines by multiplex analysis in conditioned medium from colonoids derived from *N* = 6 donors in response to different stimulation and/or treatments (1–6) at both 2% and 20% oxygen concentrations. The violin plot shows the average expression levels of chemokines (pg/ml) significantly regulated across at least one treatment contrast as indicated. The colonoids were pre-treated with tofacitinib or budesonide for 3 days alone or before TNF + Poly(I:C) stimulation (see Fig. 1). Some of the results from TNF + Poly(I:C) stimulation alone have previously been published as a comparison between culturing colonoids in 2% vs. 20% oxygen^17^. P-values were adjusted using Benjamini-Hochberg method for multiple comparisons testing. *P < 0.05, **P < 0.001, ***P < 0.0001. (**c** – left panel) CXCL2, CXCL5, and CXCL11 chemokines in conditioned media were detected by ELISA. Violin plots show concentration (pg/ml) of CXCL2, CXCL5, and CXCL11 in response to different treatments (1–6), and oxygen concentrations. Differences were evaluated using RM one-way ANOVA. (**c** – right panel) Paired analysis of CXCL2, CXCL5, and CXCL11 concentrations (pg/ml) in conditioned medium from TNF + Poly(I:C) (4) vs. tofacitinib or budesonide + TNF + poly(I:C) (5 or 6). Differences were evaluated using paired t-tests. *P < 0.05, **P < 0.01, ***P < 0.001, ****P < 0.0001.

Chemokine release is an important immunoregulatory characteristic of the intestinal epithelium^2,34^, and pro-inflammatory stimuli are shown to induce the release of chemokines from colonoids^16,17,35,36^. Thus, we did a multiplex screening featuring 40 cytokines on conditioned medium to investigate whether IBD-related chemokine release was altered from colonoids pre-treated with tofacitinib or budesonide alone or before TNF + Poly(I:C) stimulation. Since we previously observed a tendency of higher expression levels of cytokines from colonoids cultured at 20% than 2% oxygen^17^, we also included results from experiments performed at 20% oxygen. Our initial analysis showed significant regulation of 24 chemokines for the drug-only LMM (Supplementary Figure S3a) and 25 for the drug + stimulation LMM (Supplementary Figure S3b). Our drug-only LMM analysis revealed differential regulation of chemokines by tofacitinib and budesonide alone. A common set of 13 chemokines were modulated by both drugs, while each drug exclusively regulated 2 chemokines. There were generally overlapping values and low range changes between the groups, but tofacitinib seemed to increase chemokine release compared to the DMSO control. In contrast budesonide tended to reduce chemokine release (Fig. 8a). From the drug + stimulation LMM, TNF + Poly(I:C) alone significantly increased 23 chemokines compared to the DMSO control (Supplementary Figure S3b). Of these, tofacitinib pre-treatment significantly reduced the release of four chemokines: CXCL2, CXCL5, CXCL10, and CX3CL1, and enhanced release of CXCL6 and granulocyte-macrophage colony-stimulating factor (CSF). Budesonide pre-treatment, on the other hand, significantly reduced only the release of CXCL5 and CSF (Fig. 8b).

To confirm data from the multiplex, we measured the release of CXCL2, CXCL5, and CXCL11 in conditioned media by more specific ELISAs (Fig. 8c). CXCL11 was above the detection limit in the multiplex data set but was chosen for verification due to a better-performing ELISA kit since CXCL11 and 10 belong to the same chemokine group^2^. Similar results were seen when looking at colonoids cultured at 20% and 2% oxygen. The results verified that tofacitinib pre-treatment significantly attenuated the release of TNF + Poly(I:C) induced CXCL2, CXCL5 and CXCL11 (P values < 0.003), while budesonide pre-treatment significantly reduced the release of CXCL2 (*P* < 0.0001) and CXCL5 (P value < 0.0003), but not CXCL11. Overall, these findings show that tofacitinib and budesonide attenuate TNF + Poly(I:C) stimulated release of some, but not all, of the IBD-related chemokines.

## Discussion

IBD is a chronic condition characterized by cycles of remission, where symptoms are minimal or absent, and relapses, where symptoms flare up. Treatments for IBD therefore include both induction therapy aimed at reducing inflammation, alleviating symptoms, and achieving remission, and maintenance therapy to sustain remission and prevent relapses^37^. Until now, our understanding of how most IBD drugs impact IECs has been limited, particularly regarding mechanisms related to promoting mucosal healing and maintaining remission. Human colonoids recapitulate important features of colonic epithelium in a donor-specific manner^17,19,38^. In this study we therefore used colonoids derived from UC patients and non-IBD healthy controls to investigate cellular effects of the targeted small molecule and pan-JAK inhibitor tofacitinib, and the broad-spectrum, first-line corticosteroid budesonide on IECs. All colonoids used were generated from biopsies taken from uninflamed area in the right (hepatic) flexure to minimize variation due to location-specific characteristics or inflammation induced imprinting in the epithelium^39^. We did not attempt to investigate differences between healthy and IBD but included colonoids from both groups and analyzed thousands of individual organoids to examine consistency of the cellular effects. We analyzed drug-effects on differentiating colonoids cultivated in uninflamed conditions, and whether the two drugs could prevent or attenuate inflammatory responses upon pro-inflammatory stimulations with TNF + Poly(I:C). Thus, the experimental protocols used in the presents study mimics maintenance/preventions treatment. We have previously shown that tofacitinib pre-treatment inhibited TNF-Poly(I:C) induced phosphorylation of STAT1 in colonoids from all the 8 donors used in the present study when cultivated at 20% oxygen^19^. Here we cultured colonoids in 2% oxygen (physioxia) as well to enhances the translational value of the model system^28^. Our earlier work have shown improved colonid growth, more consistent and donor-dependent responses that strongly indicated that a more closely resemblance to in vivo conditions is of essence in IBD-related experimental^16,17^.

Most previous studies of tofacitinib and corticosteroids on human IECs have focused on short-time inhibitory effects and found reduced cytokine release, barrier dysfunction, and cell death^13–15,40–44^. There are not many other studies looking at growth, but corticosteroids have been shown to reduce the size of both mouse and human small intestinal organoids^45^ (enteroids). Studies in preclinical animal models also showed that corticosteroids negatively impact epithelial proliferation and wound healing^46^. Jang et al. reported unchanged morphology of human colonoids after exposure to increasing doses of tofacitinib during differentiation, but systematic measurements were not performed^47^. Further, the effects on IECs’ growth and stemness relevant for homeostasis as well as regeneration are mainly unknown. Thus, this study investigated the effect on colonoid growth and maturation when the drugs were present for a longer period from the initiation of differentiation. Using brightfield images and Fiji, a comprehensive analysis of the largest 600–1200 colonoids per condition for each donor (*N* = 8) showed that tofacitinib treatment led to an increase, and budesonide decreased colonoid size. There were large inter-individual differences between the donors. This variability might reflect the heterogenic drug responses seen in IBD patients^6,7,37^. Recently, it was shown that patient-derived intestinal organoids could predict clinical responsiveness to tofacitinib treatment in vivo^47^. The authors reported that tofacitinib inhibited pSTAT1 in tofacitinib sensitive UC organoids, but not in organoids insensitive to tofacitinib. This finding was related to tofacitinib uptake by the organic cation transporter MATE1 (SLC47A1). We did not analyse tofacitinib uptake in our colonoids, but as mentioned, previous findings support that our colonoids are tofacitinib responsive^19^. Although inter-individual differences may reflect variations in drug uptake or sensitivity, the cellular response on colonoid size and stemness during differentiation appeared robustly significant across independent experiments and analysis performed.

In addition to using Fiji for the automated size measurements of colonoids, we employed digital image analysis with QuPath to quantify all the immunostainings. We have trained and optimized scripts for each analysis, aiming to enhance the efficiency and accuracy of quantification while minimizing subjective bias. Interestingly, we found that the enlarged size induced by tofacitinib was associated with increased proliferation in general and specifically in terminally differentiated CK20-expressing cells. A recent study from Maciag et al.^48^ suggests that CK20-expressing epithelial cells can revert to a proliferative state when placed in a growth-permissive environment, both in vivo in UC patients and in genetically engineered human organoid KRT20 dT Tomato reporter line. Adding IL22 during the first 72 h enhanced organoid formation efficiency and size of the KRT20-expressing reporter line by activating the JAK/STAT pathway and upregulating REG1A. This improvement was blocked when tofacitinib was added concurrently with IL22. The cytokine IL22 activation of STAT3 phosphorylation has been shown to promote intestinal stem cell mediated epithelial regeneration in mice, and formations of human enteroids *ex vivo.*^49^ It is also reported that activation of slowly cycling reserve intestinal stem cells, which is crucial for intestinal regeneration following acute inflammation in mice, requires the JAK/STAT1 signaling pathway^50^. In contrast, we found that tofacitinib added during colonoid differentiation retained higher protein expression of stem cell markers, especially LGR5. Of note, tofacitinib seemed to retain the markers also under inflammatory stimulation with TNF + Poly(I:C), and inflammation is shown to reduce stemness^30^. This finding is consistent with an abstract reporting increased proliferation, stemness, and expansion of mice intestinal organoids by blocking the JAK/STAT pathway in both normal and TNF-stimulated conditions^51^. Pennel et al. observed high Ki67 expression in cancer-adjacent normal colon tissue treated with tofacitinib but reduced expression in tumor tissue^52^. Similarly, mice colonic organoids stimulated for 24 h with 1 µM tofacitinib exhibited a tendency towards increased proliferation^53^.

pSTAT1 is reported to induce cell cycle arrest and cell death^54^. We have previously shown that tofacitinib downregulate TNF + Poly(I:C) induced pSTAT1^19^. While e.g., IL22 induced STAT3 activation has been shown to promote proliferation in stem cells^49^, activated STAT1 may inhibit proliferation in differentiated cells by inducing expression of cyclin-dependent kinase inhibitors such as p21WAF and p27KIP, which halt the cell cycle in the G1 phase^54^. Thus, the effects of JAK inhibition on cell proliferation may be both cell and context dependent^9^. It would be intriguing to examine the effects of tofacitinib exposure alone or with other pro-inflammatory cytokines during the growth phase of human colonoids. Future in vitro studies should also examine mechanistic drug effects in rescue protocols where cellular responses to inflammatory cytokines are already present. Budesonide is a drug used to induce remission in UC. However, in an in vitro setting, a protocol for inducing remission is challenging to implement because adding a proinflammatory stimulus triggers a cascade of inflammatory signals, potentially overwhelming the effects of anti-inflammatory drugs. Also, induction/rescue therapy may depend more on the interactions between the immune system and IECs, which is harder to replicate in the organoid model compared to adding relevant components in a pre-treatment protocol or administering drugs simultaneously with pro-inflammatory cytokines. Therefore, additional pre-clinical models are necessary to support the use of patient-derived organoid models for this type of research^39,55,56^. Nonetheless, our findings show that budesonide attenuates inflammatory signals from IECs, suggesting that it also can limit ongoing inflammation as observed in clinical use.

Adding tofacitinib during the colonoid differentiation process appears to change the behavior patterns of the IECs, promoting epithelial cells with the same degree of maturation but with a retained regenerative and self-renewal potential. The same was not seen with budesonide, which resulted in reduced colonoid size without consistently affecting the proliferation ability of IECs. The response to tofacitinib was more consistent than to budesonide, fitting with the broad-spectrum nature of corticosteroids. Still the opposing effects of the two drugs were obvious. The different impacts of tofacitinib and budesonide are relevant to their ability to promote mucosal healing during inflammation and for tofacitinib the impact on epithelial stemness is relevant to maintain homeostasis during remission. There has been some concern related to cancer risk in patients treated with tofacitinib^57,58^. Thus, understanding how tofacitinib affects cell growth and stem cell properties in various cell types could aid in assessing risks during different disease phases or design treatments to minimize unintended impacts on cellular responses.

Increased IEC death has been reported to be a prevalent pathological characteristic of IBD and is induced by inflammatory processes involving, e.g., TNF and TLR3 ligands (like Poly(I:C))^59,60^. Previous studies have reported that both tofacitinib and budesonide can protect IEC from cytokine-mediated barrier dysfunction or cell death^13–15,44^, but to our knowledge, effects on the cell death types apoptosis and necroptosis have not been thoroughly examined in human colonoids. In our experiments, TNF + Poly(I:C) stimulation for 24 h increased expression of the apoptosis marker cleaved caspase 3 and the necroptosis marker pMLKL, but neither tofacitinib nor budesonide pre-treatment for 3 days had protective effects. Interestingly, our data show that treatment with tofacitinib alone increased pMLKL expression (but not cleaved caspase 3), which tended to increase even more when combined with TNF + Poly(I:C) stimulation. This regulation by tofacitinib and the possible implications should be further evaluated.

Previous work from our group^16,32^ and others^34,61^ has emphasized the importance of chemoattractant signaling pathways in the gut, which originates with epithelium. Multiplex chemokine analysis showed that tofacitinib and budesonide had mainly opposite effects in uninflamed colonoids. Since the concentrations of cytokines in conditioned medium were generally higher in tofacitinib-treated colonoids and lower in budesonide treated colonoids compared to untreated controls, this finding may reflect the drugs effect on colonoid size. Nevertheless, pre-treatment with tofacitinib and budesonide attenuated TNF + Poly(I:C) induced release of the neutrophile chemoattractants CXCL2 and CXCL5, but only tofacitinib reduced release of the T-cell chemoattractants CXCL10/11 and CX3CL1^2^. Interestingly, tofacitinib increased while budesonide decreased release of CSF also in TNF + Poly(I:C) stimulated colonoids. CSF release from IECs may regulate crypt cell proliferation^62–64^. However, further research is necessary to determine if variations in CSF release merely reflect differences in organoid size or if CSF actively regulate the growth.

In summary, the patient-derived organoids model has been utilized to investigate the effects of tofacitinib and budesonide on IEC stemness, growth, cell death, and chemokine release. Our main finding is that adding the pan JAK-inhibitor tofacitinib during differentiation promotes stemness and a heightened tendency for proliferation in terminally differentiated colonoids. To our knowledge, this is the first study examining these effects on IECs, using computational analyses of numerous colonoids from several donors. Although both budesonide and tofacitinib can attenuate chemokine release from IECs, their effects on other parameters important for epithelial homeostasis differ and may have large inter-individual variations. Further research is needed to understand more about how signaling pathways are affected by tofacitinib and budesonide in IECs. However, our study demonstrates that patient-derived colonoids can be used to investigate MOA in IECs for different classes of IBD drugs and identify differences on the level of an individual that may be relevant for personalized IBD treatment. In the context of disease, enhanced stemness may promote repair and regeneration of the mucosal lining. Thus, drug delivery specifically to mucosal epithelium could provide a better ability to treat IBD-patients.

## Electronic supplementary material

Below is the link to the electronic supplementary material.

Supplementary Material 1

Supplementary Material 2

## Data Availability

All data generated or analysed during this study are included in the two Supplementary files which present raw data, full blot images and quantification scripts.

## Abbreviations

CK20: Cytokeratin 20
CSF: Granulocyte-macrophage colony-stimulating factor
DAB: 3, 3’-diaminobenzidine
DAPI: 4’,6-diamidino-2-phenylindole
DMSO: Dimethyl sulfoxide
EPHB2: EPH Receptor B2
FFPE: Formalin-fixed paraffin embedded
GAPDH: Glyceraldehyde-3-phosphate dehydrogenase
HC: Healthy control
IBD: Inflammatory bowel disease
IEC: Intestinal epithelial cell
JAK: Janus kinase
LGR5: Leucine-rich repeat-containing G-protein coupled receptor 5+
MHC-II: Major histocompatibility complex-II
MOA: Mechanisms of action
PBS: Phosphate-buffered saline
pMLKL: Phosphorylated mixed lineage kinase domain-like protein
poly(I:C): Polyinosinic:polycytidylic acid
RM: Repeated measurement
STAT: Signal transducer and activator of transcription protein
TLR3: Toll-like receptor 3
TNF: Tumor necrosis factor
TUNEL: Terminal deoxynucleotidyl transferase-mediated dUTP nick end labeling
UC: Ulcerative colitis
