# Supplementary material for: Tofacitinib and budesonide treatment affect stemness and chemokine release in IBD patient-derived colonoids
Source: Sci Rep. 2025 Jan 30;15:3753. doi: 10.1038/s41598-025-86314-2 (PMC11782514; doi:10.1038/s41598-025-86314-2)
Supplement: Supplementary file 2 — Supplementary Material 2 [file 41598_2025_86314_MOESM2_ESM.pdf]

Supplementary Table 1

Name	Conc.	Cat#	Manufacturer
**Ligands and reagents for colonoid culturing and stimulation***			
Nicotinamide	1221.2µg/mL	N3376-100G	MerckMillipore
SB202190	3.31µg/mL	S7067	Sigma-Aldrich
DAPT	4.3µg/mL	2634	Bio-Techne
Matrigel® Growth Factor Reduced (GFR) Basement Membrane Matrix, phenol red-free	100 cells/µL	734-1101	Corning®
Richard-Allan Scientific™ HistoGel™ Specimen Processing Gel		HG-4000-012	Thermo Scientific
Recombinant human Tumor necrosis factor-α (TNF)	100 ng/mL	300-01A	PeproTech
Polyinosinic:polycytidylic acid (Poly(I:C))	20 µg/mL	tlrl-pic	InvivoGen
Tofacitinib Citrate (Tofacitinib)	50 µM	S5001, CP-690550	Selleckchem.com
Budesonide	10 µM	S1286	Selleckchem.com
Wnt-3A conditioned medium	50% for growth media and 5% for differentiation media	CRL-2647	ATCC
**Western blot reagents**			
1x Complete® EDTA-free protease inhibitor	1X	11836170001	Roche Life Science Products
Utrapure EDTA (0.5M)	5nM	15575-038	Thermo Scientific
NP-40	1 %	492018	Sigma-Aldrich
Phosphatase inhibitor cocktail 2 (PIC2)	1X	P5726	Sigma-Aldrich
Phosphatase inhibitor cocktail 3 (PIC3)	1X	P0044	Sigma-Aldrich
4-12% Nupage Bis-Tris gel		NP0321BOX	Thermo Scientific
Trans-Blot Turbo nitrocellulose membrane 0.2 µm		1704158	Bio-Rad laboratories
NuPage LDS Sample buffer (4X)	1X	NP0007	Thermo Scientific
Blocking Buffer for fluorescent Western blot		MB-070	Rockland Immunochemicals
**Staining Kits and associated reagents**			
EnVision FLEX+, Mouse, High pH		K8012	Dako Agilent
MaxDouble M488&R650 ImmunoFluorescence Double Staining Kit for human tissue		DSMR-H3	MaxVision Biosciences Inc.

In Situ Cell Death Detection Kit, Fluorescein		11684795910	Roche Life Science Products
Pierce BCA protein assay kit		23225	Thermo Scientific
Proteinase K	20 µg/mL	AM2546	Thermo Scientific
DAPI	1:1000	62248	Thermo Scientific
**Antibodies**			
CK20, mouse mAb, clone Ks20.8	1:1000 auto / 1:150 manual staining	M7019, RRID:AB_2133718	Dako Agilent
Ki67 mouse mAB, clone MIB-1	1:600 auto /1:50 manual staining	M7240, RRID:AB_2631211	Dako Agilent
LGR5 mouse mAb, clone OTI2A2	1:1000	MA5-25644, RRID:AB_2723318	Thermo Scientific
EphB2 goat pAb	1:1000	AF467, RRID:AB_355375	R&D Systems
Phospho-MLKL (Ser358) rabbit mAb, clone E7G7P	1:1000	18640S	Cell Signaling Technology
MLKL mouse mAb, clone E7v4W	1.1000	26539	Cell Signaling Technology
Caspase-3 rabbit mAb, clone D3R6Y (also recognizing cleaved caspase-3)	1:1000	14220, RRID:AB_2798429	Cell Signaling Technology
GAPDH XP rabbit mAb, clone D16H11	1:5000	5174, RRID:AB_10622025	Cell Signaling Technology
Goat anti-mouse IgG, DyLight800	1:5000	SA5-35521, RRID:AB_2556774	Thermo Scientific
Goat anti-mouse IgG, DyLight680	1:5000	35518, RRID:AB_614942	Thermo Scientific
Goat anti-rabbit IgG, DyLight800	1:5000	SA5-35571, RRID:AB_2556775	Thermo Scientific
Goat anti-rabbit IgG, DyLight680	1:5000	35569, RRID:AB_1965957	Thermo Scientific
Donkey anti-goat Donkey anti-Goat IgG, Alexa Fluor™ 647	1:5000	A-21447, RRID:AB_2535864	Thermo Scientific
**Multiplex and ELISA kits**			
Bio-Plex Pro Human Chemokine Panel, 40-plex		171ak99mr2	Bio-Rad laboratories
Human CXCL2/GRO beta DuoSet ELISA		DY276-05	R&D systems
Human CXCL5/ENA-78 DuoSet ELISA		DY254-05	R&D systems
Human CXCL11/I-TAC DuoSet ELISA		DY672	R&D systems
**Softwares**			

Fiji	RRID:SCR_002285, Schindelin et al., 2012
QuPath 3.0	RRID:SCR_018257, Bankhead et al., 2017
Rstudio	RRID:SCR_001905
Graphpad Prism 9	RRID:SCR_002798, GraphPad Software Inc.
LI-COR Odyssey Fc Imager, v3.0.25	RRID:SCR_023227, LI-COR Biotechnology
LI-COR Image Studie Lite, v3.1	RRID:SCR_013715, LI-COR Biotechnology

*Comprehensive protocol in Gopalakrishnan, S., et al., *Comprehensive protocols for culturing and molecular biological analysis of IBD patient-derived colon epithelial organoids.* Frontiers in Immunology, 2023. **14**.

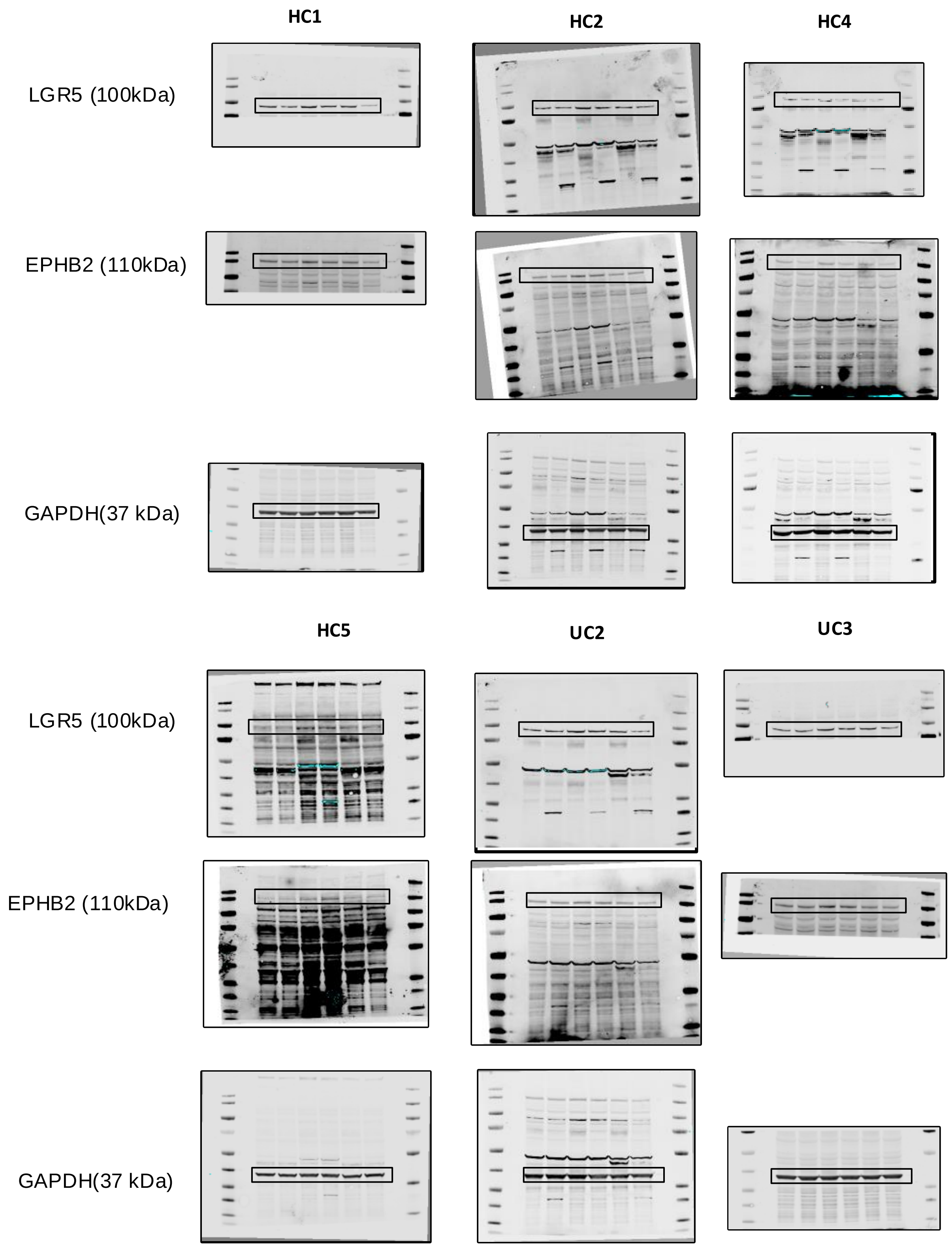

Figure S1. Full blot images with boxes indicating the respective immunoblot bands shown in Figure 6

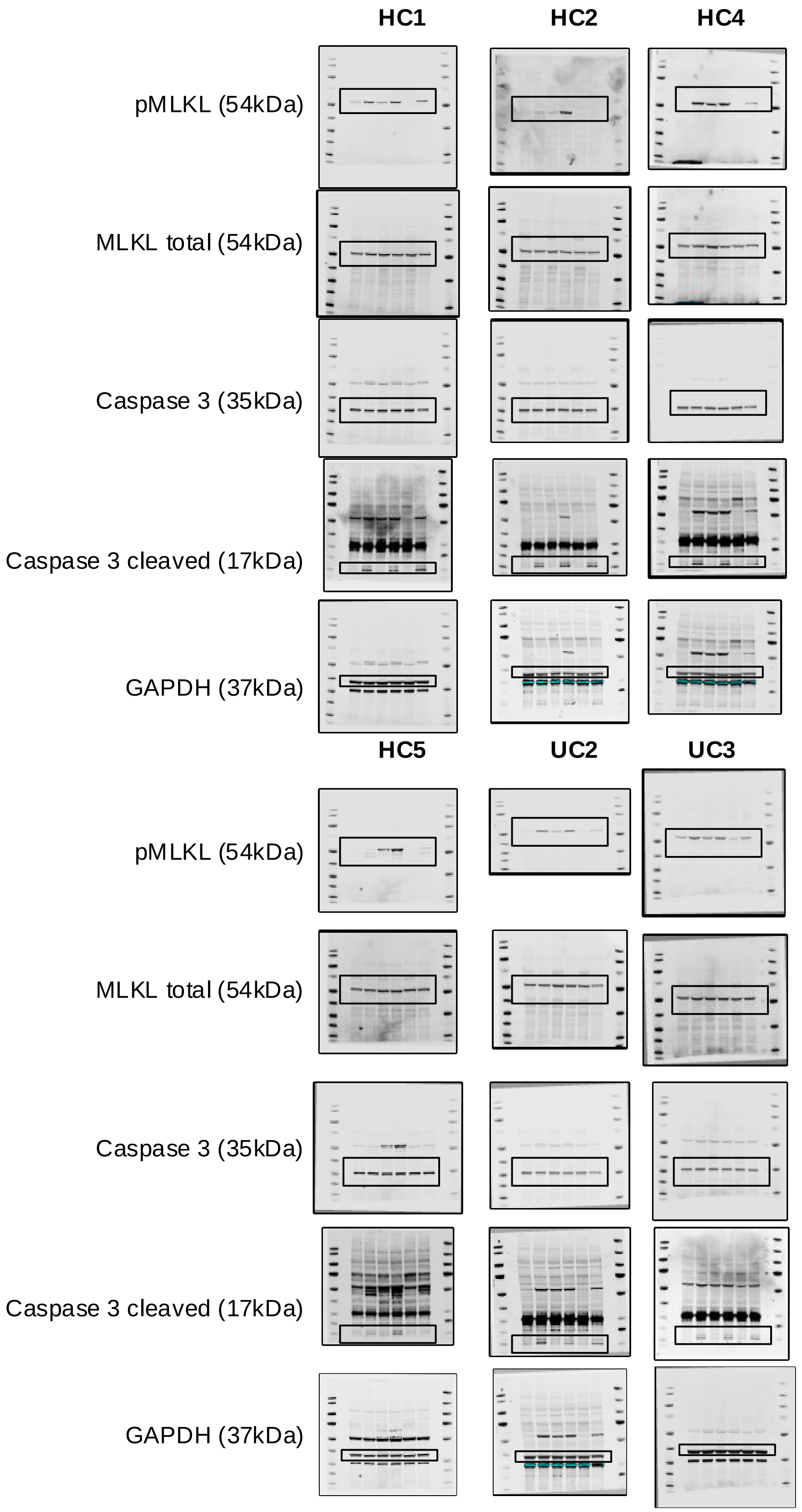

Figure S2. Full blot images with boxes indicating the respective immunoblot bands shown in Figure 8.

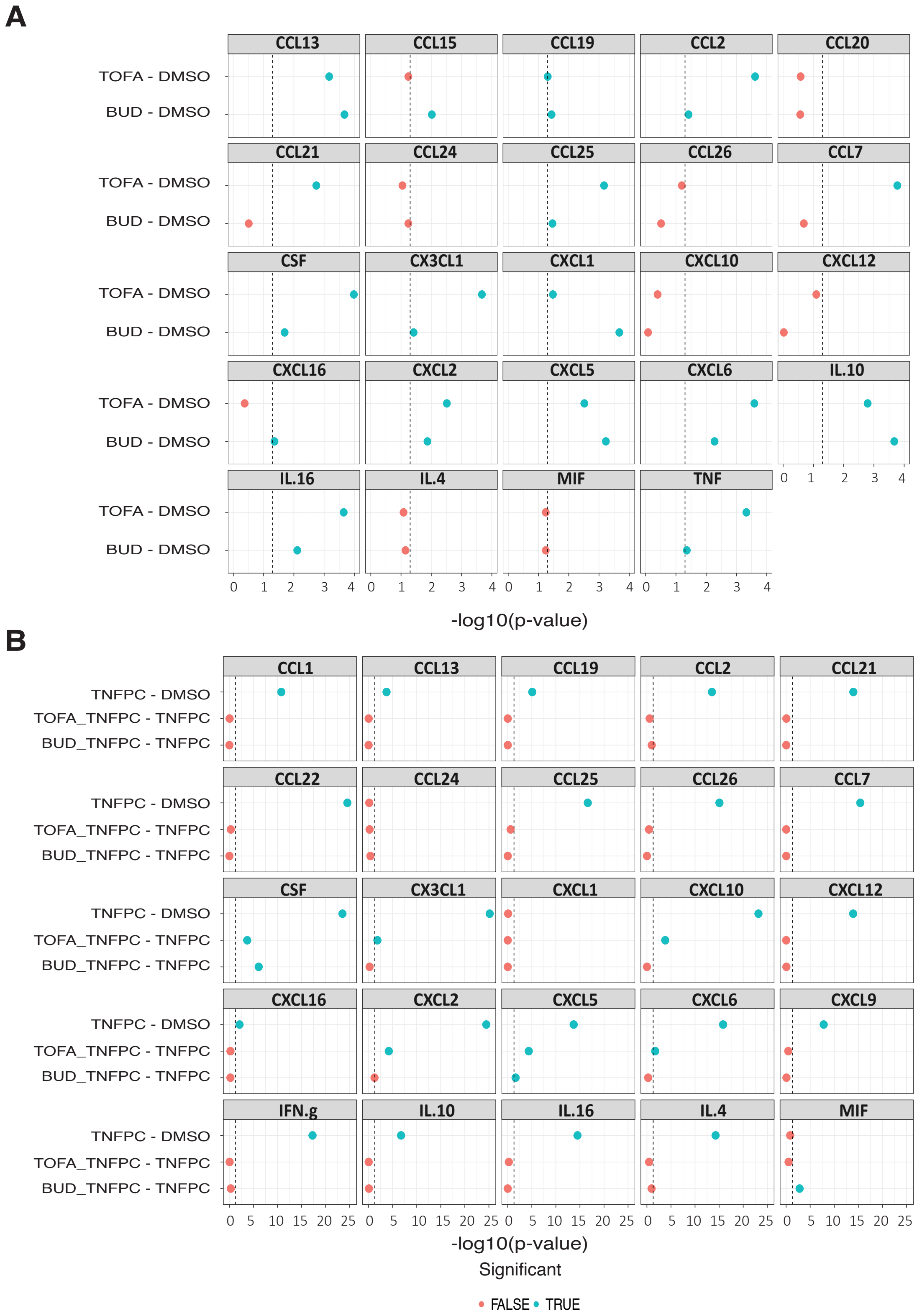

Figure S3. Panels in A and B show result from the linear mixed model (LMM) used to evaluate significance across different contrasts. Each panel represents a distinct cytokine, with the X-axis indicating different comparisons and the Y-axis representing the negative log10 of adjusted p-values. A horizontal dashed line at -log10(0.05) represents the typical threshold for statistical significance, and points above these lines are considered statistically significant (blue dots). P-values were adjusted using Benjamini-Hochberg method for multiple comparisons testing. (TOFA- tofacitinib, BUD- budesonide, TNFPC-TNF+Ploy(I:C))
